# The Effect of Topical Application of Royal Jelly on Chemoradiotherapy-Induced Mucositis in Head and Neck Cancer: A Preliminary Study

**DOI:** 10.1155/2014/974967

**Published:** 2014-10-21

**Authors:** Kohichi Yamauchi, Yasunao Kogashiwa, Yorihisa Moro, Naoyuki Kohno

**Affiliations:** Department of Otolaryngology, Head and Neck Surgery, Kyorin University School of Medicine, 6-20-2 Shinkawa, Mitaka, Tokyo 181-8611, Japan

## Abstract

*Purpose*. One of the common side effects experienced by head and neck cancer patients on chemoradiotherapy is mucositis. Severe mucositis may be controllable by limiting cancer therapy, but it has resulted in decreasing the completion rate of chemoradiotherapy. The efficacy of royal jelly (RJ) as prophylaxis against chemoradiotherapy-induced mucositis was evaluated through clinical scoring of oral and pharyngeal mucositis. *Methods*. In this randomized, single-blind (physician-blind), clinical trial, 13 patients with head and neck cancer requiring chemoradiation were randomly assigned to two groups. Seven patients assigned to the study group received RJ, and 6 patients were assigned to the control group. RJ group patients took RJ three times per day during treatment. The patients in both groups were evaluated twice a week for the development of mucositis using Common Terminology Criteria for Adverse Events version 3.0. *Results*. A significant reduction in mucositis was seen among RJ-treated patients compared with controls (*P* < 0.001). *Conclusion*. This study demonstrated that prophylactic use of RJ was effective in reducing mucositis induced by chemoradiotherapy in head and neck cancer patients. However, further studies are needed because of the small sample size and the absence of double blinding.

## 1. Introduction

Chemoradiotherapy for head and neck cancer induces mucositis, and it can also cause ulcers. Patients may experience pain, dysphagia, and dysphonia. As patients lose their oral feeding ability, they require external nutrition support. Strong early side effects of chemoradiotherapy may be controllable by limiting cancer therapy, but this has resulted in decreasing the chemoradiotherapy completion rate.

Many authors have reported the prophylactic use of bee products such as honey, royal jelly (RJ), and propolis for oral mucositis [1–7]. Kohno et al. reported the prophylactic use of honey extract as concurrent chemoradiotherapy for head and neck cancer patients [8]. Suemaru et al. also evaluated their effects on 5-fluorouracil-induced experimental oral mucositis in hamsters [9]. Erdem and Gungormus evaluated the effect of RJ on oral mucositis in patients undergoing radiotherapy and chemotherapy, and they reported that the mean time to resolution of oral mucositis was significantly shorter in the RJ group than in the control group [10].

The results suggested that the topical application of royal jelly may have a healing effect on severe oral mucositis induced by chemotherapy. Therefore, the efficacy of RJ as prophylaxis against chemoradiotherapy-induced mucositis was evaluated.

## 2. Materials and Methods

### 2.1. Design

The objective of this study was to evaluate the efficacy of RJ as prophylaxis against chemoradiotherapy-induced mucositis through clinical scoring of oral and pharyngeal mucositis in head and neck cancer patients.

This study was approved by the Ethics Committee of Kyorin University. All patients provided their written, informed consent. Head and neck squamous cell carcinoma patients were enrolled. Eligible patients were aged >18 years with a performance status of 0 to 1. Patients were randomly assigned to the control and RJ groups in this single-blind (physician-blind), clinical trial.

### 2.2. Induction Chemotherapy

The regimen was as follows. Nedaplatin (80 mg/m^2^) was administered on day 1, and S-1 was simultaneously administered to patients orally twice daily at an initial dose of 65 mg/m^2^/day (patients with body surface area (BSA) > 1.5 m^2^ received 100 mg/day; patients with 1.25 m^2^ < BSA < 1.5 m^2^ received 80 mg/day) for 2 weeks (days 1–14).

### 2.3. Concomitant Chemotherapy and Radiotherapy

Three chemotherapy regimens were used.

*Weekly Nedaplatin and Docetaxel Regimen*. During radiotherapy, weekly nedaplatin (15 mg/m^2^) and docetaxel (10 mg/m^2^) were administered for 6 courses intravenously.

*S-1 Regimen*. S-1 was administered at a dose of 80 mg/day on alternate days for 6 weeks.

*Cisplatin Regimen*. This was a form of selective arterial chemotherapy. Cisplatin (5 mg/m^2^) was administered with the catheter in the vessel feeding the tumor on Monday to Friday (5 days/week) for 6 weeks.

Radiotherapy was administered to both groups: 2.0 Gy/day fractions on Monday to Friday for 33 to 35 fractions, for a total dose of 66 to 70 Gy by Linac.

### 2.4. RJ Group

RJ was prepared by Yamada Apiculture Center, Inc. (Okayama, Japan). RJ was collected from* Apis mellifera *L. that fed primarily on nectar and pollen from several flowers in Zhejiang, China. This product complies with the organic standards of the European Union. The product name is “organic royal jelly-gen nyu.” It has the consistency of an ointment and contains a 1 gram measuring spoon that patients used to apply the RJ. The RJ group took 1 gram of RJ three times a day (3 g/day) during radiation treatment.

### 2.5. Control Group

The control group did not take any RJ.

### 2.6. Evaluation

Evaluation was done during the radiation period and 1 month after radiation. Patients were evaluated twice a week from the mouth to the pharynx by inspection and fiberscope examination. The reaction of the mucosa was graded using the Common Terminology Criteria for Adverse Events version 3.0 (CTCAE). The mucositis was graded as follows: Grade 1, erythema of the mucosa; Grade 2, patchy ulcerations or pseudomembranes; Grade 3, confluent ulcerations or pseudomembranes, bleeding with minor trauma; Grade 4, tissue necrosis, significant spontaneous bleeding, life-threatening consequences; and Grade 5, death.

### 2.7. Statistical Analysis

Data are shown as means ± standard deviation. Statistical significance was analyzed using the nonparametric Mann-Whitney *U* test for 2 groups. *P* < 0.05 was considered significant.

## 3. Results

### 3.1. Patients' Characteristics

This study was done at Kyorin University Hospital, Japan, between 2009 and 2010. Thirteen patients (12 males, 1 female; median age 65.0 years; age range, 51–84 years) diagnosed with head and neck cancer were enrolled in the trial. The primary cancer site was hypopharyngeal in 5 patients, oropharyngeal in 4, laryngeal in 2, oral cavity in 1, and maxillary sinus in 1. The patients' characteristics are shown in Table 1. Seven patients were assigned to the RJ group and 6 to the control group.

There were no side effects such as allergy, irritation, or toxicity during treatment. All patients of both groups completed planned chemoradiotherapy.

### 3.2. Mucositis

Four patients received induction chemotherapy. All had no sign of mucositis at the beginning of concomitant chemoradiotherapy.

At the end of radiation, in the RJ group, Grade 3 mucositis was observed in 71.4% (5/7), and Grade 2 was seen in 28.6% (2/7). In the control group, Grade 3 mucositis was seen in 100% (6/6). In the control group, one case progressed to Grade 4 one month after treatment.

**Figure 1:**
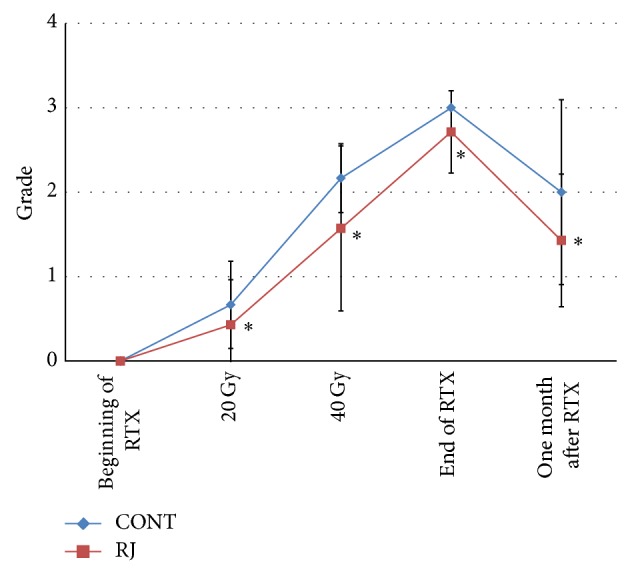
Grades of mucositis. Each data point represents the mean ± SD. Mann-Whitney *U* test: ^*^ *P* < 0.001 versus control. RTX: radiotherapy.

Figure 1 shows the grades of mucositis at the beginning of radiation, after 20 Gy, after 40 Gy, at the end of radiation, and 1 month after radiation. A significant difference was observed between the groups in the grade of mucositis at each point.

**Figure 2:**
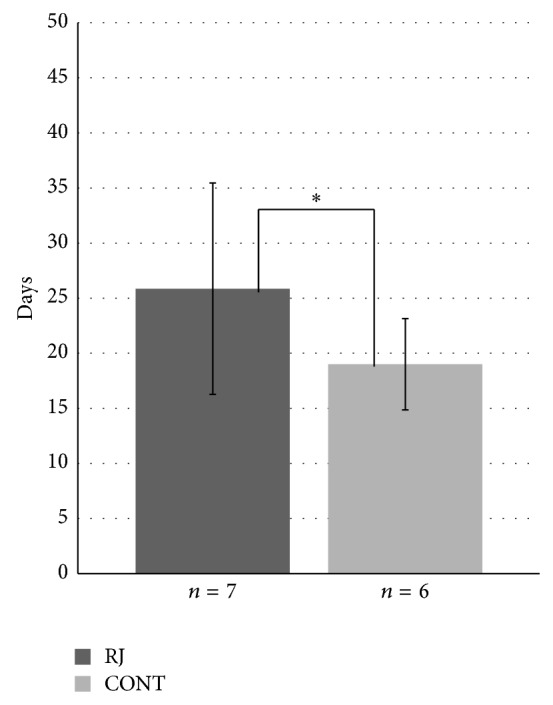
Time to progress to G2 mucositis. Each data point represents the mean ± SD. Mann-Whitney *U* test: ^*^ *P* < 0.001 versus control.

Figure 2 shows the average time to progress to Grade 2 mucositis from the beginning of radiation in both groups. The average time was 25.9 ± 9.6 days in the RJ group and 19.0 ± 4.1 days in the control group. The average time was significantly longer in the RJ group (*P* < 0.001).

**Figure 3:**
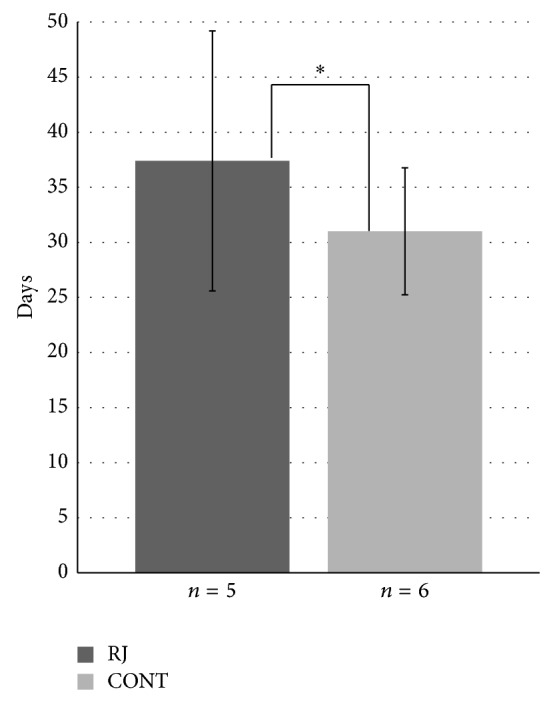
Time to progress to G3 mucositis. Each data point represents the mean ± SD. Mann-Whitney *U* test: ^*^ *P* < 0.001 versus control.

Figure 3 shows the average time to progress to grade 3 mucositis from the beginning of radiation in both groups. The average time was 37.4 ± 11.8 days in the RJ group and 31.0 ± 5.8 days in the control group. The average time was significantly longer in the RJ group (*P* < 0.001).

The results shown in Figures 1 and 2 suggest that applying RJ may prevent or reduce the severity of chemoradiotherapy-induced mucositis.

## 4. Discussion

Oral mucositis occurs in 15–40% of patients receiving standard chemotherapy, and 100% of patients receiving radiation therapy for head and neck cancer develop oral mucositis of varying degrees [11, 12]. The mechanism by which mucositis occurs is based on the fact that the oral mucosa has a high level of mitotic activity and high cell turnover. Due to the high degree of cell desquamation, there is a continuous need for cell multiplication to recover the oral mucosa. Tissues with high levels of mitotic activity respond rapidly to radiation, since the most sensitive phases of the cell cycle are G2 and mitosis. Thus, the mucosa is rapidly affected [13]. The same is true for chemotherapeutic drugs such as cisplatin, S-1, nedaplatin, and docetaxel. Chemoradiotherapy causes various changes in normal tissues, depending on the closely interrelated factors of total dose, fractionation schedule, and volume treated. Until now, there has been no way to prevent chemoradiotherapy-induced mucositis using only gargling and analgesics.

Bee products are commonly used traditionally to treat not only mucositis, but also skin disorders like cuts and burns as traditional wound healing agents [14–17]. Bee products are not artificial, but natural resources. This point results in easy acceptance among many cancer patients because they must always take so many artificial medicines. Therefore, this study evaluated the efficacy of bee products for mucositis induced by chemoradiotherapy.

The first issue that needed to be resolved was which kind of bee product would be best for mucositis. Suemaru et al. already evaluated three bee products (honey, RJ, and propolis) for 5-fluorouracil-induced experimental oral mucositis in hamsters [9]. They reported that only the RJ ointments significantly improved recovery from chemotherapy-induced mucositis in a dose-dependent manner. These results suggested that topical application of RJ has a healing effect on severe oral mucositis induced by chemotherapy. This is the reason why RJ was selected for this study.

RJ is mainly secreted by the hypopharyngeal and mandibular glands of worker honeybees between the sixth and twelfth days of their life, and it is an essential food for the development of the queen honeybee. RJ is a complex substance containing a unique combination of proteins (12–15%), sugars (10–12%), lipids (3–7%), amino acids, vitamins, and minerals [18]. RJ has also been demonstrated to possess many pharmacological activities in experimental animals, including antitumor [19], antioxidant [20, 21], anti-inflammatory [22], antibacterial [23], antiallergic [24], antiaging [25], and antihypertensive properties [26]. Recently, many authors have reported that the antioxidant effect is important for wound healing [27–29]. In this respect, RJ is an ideal agent. Inoue et al. reported the effect of dietary RJ on tissue DNA oxidative damage in mice [20]. In mice that were fed a dietary supplement of RJ, the levels of a marker of oxidative stress, 8-hydroxy-2-deoxyguanosine, were significantly reduced in kidney DNA and serum.

Furthermore, Kohno et al. suggested that RJ has anti-inflammatory actions through inhibiting proinflammatory cytokine production by activated macrophages [22]. They named the factor honeybee RJ-derived anti-inflammatory factor.

Watanabe et al. reported that RJ showed scavenging activity for 1,1-diphenyl-2-picrylhydrazyl (DPPH) radicals, superoxide radicals, and hydroxylradicals. Therefore, in the healing effect of RJ on mucositis, radical scavenging activity is more important than keratinocyte growth factor generation [30].

RJ also has antibacterial actions. Royalisin found in the RJ of* Apis mellifera* is an antimicrobial peptide. It plays an important role in protecting wounds from being infected [23].

The present study showed that RJ prevented progression of mucositis from the early phase, and the average time to progress to Grade 2 mucositis was 25.9 ± 9.6 days versus 19.0 ± 4.1 days (RJ versus control). The average time to progress to Grade 3 was 37.4 ± 11.8 days versus 31.0 ± 5.8 days (RJ versus control). A significant reduction in mucositis occurred among RJ-treated patients compared with controls (*P* < 0.001).

At the end of radiation, Grade 3 mucositis was observed in 71.4% (5/7) in the RJ group and 100% (6/6) in the control group. These results suggest that topical application of RJ is effective in preventing accelerated mucositis induced by chemoradiotherapy.

In this study, only RJ was evaluated for mucositis, but Nakajima et al. reported that, of all bee products, propolis is the most powerful antioxidant [21]. An antioxidant effect is important in the mucositis healing process. Although we would have liked to evaluate propolis, propolis extracted with water was not available. Propolis extracted with ethanol is not good for mucositis due to stimulation by alcohol. In the future, we would like to evaluate propolis extracted with water.

Thus, further studies are needed to evaluate the effect of RJ on mucositis and elucidate the precise mechanisms of action. Nevertheless, it is possible to say that RJ tends to prevent progression of mucositis.

## Figures and Tables

**Table 1:** Patients' profile.

Case	Group	Tumor site	Sex	Age (year)	TNM stage	IC	CRT
1	RJ	Larynx	M	59	T2N0M0	—	S-1
2	RJ	Oropharynx	M	84	T3N2cM0	—	S-1
3	RJ	Hypopharynx	M	62	TXN3M0	S1 + N	N + T
4	RJ	Hypopharynx	M	58	T1N3M0	S1 + N	N + T
5	RJ	Larynx	F	75	T2N0M0	—	S-1
6	RJ	Oropharynx	M	65	T4aN2bM0	—	CDDP
7	RJ	Hypopharynx	M	51	T2N2bM0	—	N + T
8	CONT	Hypopharynx	M	84	T2N0M0	—	S-1
9	CONT	Oropharynx	M	63	T1N2bM0	—	N + T
10	CONT	Oropharynx	M	62	T1N2aM0	—	CDDP
11	CONT	Oropharynx	M	65	T4aN0M0	S1 + N	N + T
12	CONT	Hypopharynx	M	66	T2N1M0	S1 + N	S-1
13	CONT	Maxillary sinus	M	78	T4aN0M0	—	CDDP

RJ: royal jelly, CONT: control, M: male, and F: female.

IC: induction chemotherapy.

CRT: chemoradiotherapy.

S1 + N: S1 + nedaplatin.

S1: S-1 regimen.

N + T: Weekly nedaplatin and docetaxel regimen.

CDDP: Cisplatin regimen.
